# A typical presentation of moxifloxacin-induced DRESS syndrome with pulmonary involvement: a case report and review of the literature

**DOI:** 10.1186/s12890-022-02064-1

**Published:** 2022-07-19

**Authors:** Yinhong Zhang, Xiaoyan Wang, Yang Cheng, Xiaofang Wang, Yunjian Zhang

**Affiliations:** Beijing Jishuitan Hospital, Fourth Clinical College of Peking University, No. 31 Xinjiekou East Street, Xicheng District, Beijing, 100035 China

**Keywords:** Case report, Drug reaction, Eosinophilia, Dress, Moxifloxacin

## Abstract

**Background:**

Drug reaction with eosinophilia and systemic symptoms (DRESS) syndrome is a kind of hypersensitivity drug reaction involving the skin and multiple internal organ systems. Moxifloxacin has rarely been reported to be a drug that is associated with DRESS syndrome. Lungs are less frequently involved in DRESS syndrome, but their involvements may herald more serious clinical processes. We present a rare typical case of moxifloxacin-induced DRESS syndrome with lungs involved. Valuable clinical data such as changes in the pulmonary imaging and pulmonary function tests was recorded. This case is important for the differential diagnosis of DRESS syndrome with lungs involved by providing clinical manifestations, CT imaging, pulmonary function tests, and biopsy pathological characteristics. The changes in pulmonary imaging and pulmonary function tests may help us understand the mechanism of DRESS syndrome further.

**Case presentation:**

We report a case of a 47-year-old woman who was treated with oral moxifloxacin for community-acquired pneumonia. The patient subsequently developed a cough, fever, liver injury, skin rash, hematologic abnormalities, and shortness of breath (SOB) followed by pharyngeal herpes and peripheral neuritis. These symptoms, clinical lab index, and CT scan of the lungs improved after the withdrawal of moxifloxacin. The probability of moxifloxacin-induced DRESS syndrome was rated as “Definite”, with 7 scores graded by RegiSCAR. A literature search was also performed with “fluoroquinolones,” “moxifloxacin,” “ciprofloxacin,” “levofloxacin,” “delafloxacin,” and “DRESS” or “drug-induced hypersensitivity syndrome (DIHS)” as the keywords that were put into PubMed. The overall pulmonary involvement was approximately 9.1% (1/11). It is a rare reported case of DRESS syndrome with pulmonary involvement induced by moxifloxacin. We summarized detailed clinical data, including pulmonary imaging and pulmonary function changes.

**Conclusion:**

This is a rare reported case of DRESS syndrome with pulmonary involvement induced by moxifloxacin. Prompt recognition and correct diagnosis can promote appropriate treatment and accelerate recovery. This case is important for us as a reference in the differential diagnosis of DRESS syndrome and helps us further understand the mechanism of DRESS syndrome.

## Background

Drug reaction with eosinophilia and systemic symptoms (DRESS) syndrome is a kind of hypersensitivity drug reaction involving the skin and multiple organs. Symptoms are usually skin rash and fever [1], accompanied by eosinophilia, atypical lymphocytosis, and multiple organ failure [2–4]. DRESS syndrome is marked by prolonged latency, and symptoms usually appear 2 to 6 weeks after the first exposure to the offending drug [3]. There will still be a long course of disease with frequent recurrence after discontinuation of the involved drugs. It is often accompanied by reactivation of a latent human herpesvirus (HHV) infection [5]. Drugs related to DRESS syndrome include but are not limited to anticonvulsants, antidepressants and antimicrobials [3]. A recently published case series proposed that 15–37% of DRESS syndrome cases may be caused by antimicrobials [6]. Sharifzadeh et al. analyzed 254 cases of antimicrobial-induced DRESS syndrome and found that 42.13% were induced by antituberculosis, 18.1% were induced by glycopeptides, and only 1.97% were induced by fluoroquinolone [7].

Lungs (8.9%) are less frequently involved in DRESS syndrome compared with the liver (86.7%) and kidney (17.8%) [8], but pulmonary involvement may herald a more severe clinical course and higher mortality [9]. In a systematic review [9], 22 cases of DRESS syndrome with pulmonary manifestations were collected, and none were credited to fluoroquinolone. The pattern of computerized tomography of pulmonary involvement has been described in the review, but information about changes in pulmonary function and alveolar lavage fluid was not mentioned. The differential diagnosis of a combination of peripheral eosinophilia, rash, and respiratory symptoms is so complicated that 50% of patients with DRESS syndrome were initially misdiagnosed and treated for infection [10]. Therefore, it is important to study the characteristics of patients with lung involved of DRESS syndrome and to recognize them to stop offending medication and improve the outcome.

Reports of allergic reactions caused by fluoroquinolones have increased, mainly due to moxifloxacin [11]. Its metabolites may react with proteins to form covalently bound proteins that can interact with the immune system [12]. The allergic mechanism of fluoroquinolones is mainly IgE and T-cell-dependent reactions. Most of them are IgE types, such as urticaria and allergic reactions. There are fewer reports on T-cell-dependent reactions, which include maculopapular exanthema [13–16], fixed drug-induced eruption [17], acute generalized exanthematic pustulosis (AGEP) [16], Stevens-Johnson syndrome (SJS) and toxic epidermal necrolysis (TEN) [18–20]. DRESS syndrome caused by fluoroquinolones is rarely reported.

A literature search was performed with “fluoroquinolones,” “moxifloxacin,” “ciprofloxacin,” “levofloxacin,” “delafloxacin,” and “DRESS” or “drug-induced hypersensitivity syndrome (DIHS)” as the keywords put into PubMed, and 11 related cases have been reported thus far (Table 1). One case was caused by levofloxacin, two cases were caused by ciprofloxacin, and eight cases were suspected to be caused by moxifloxacin. Pulmonary involvement is very rare estimated as 9.1% (1/11), with only one case reported by Son CH et al. [21], in which the specific CT images were not shown.

**Table 1:** The different cases of DRESS syndrome associated with quinolones and compared with our case

Author	Age/sex	Drug(s) involved	Initial symptom	Organ system involved	Treatment and outcome
Alkhateeb et al. [22]	24/F	Ciprofloxacin	Fever, rash, diarrhea, muscular pain	pleural effusion	No Corticosteroids, survival
Charfi et al. [23]	26/M	Levofloxacin	Rash	Liver	No Corticosteroids, survival
Artukovic et al. [24]	57/F	Ciprofloxacin	Rash, fever	Liver	No Corticosteroids, survival
Miyagui-Namikawa et al. [25]	60/F	Moxifloxacin	Fever, rash	Liver	Corticosteroids, survival
Um et al. [26]	52/F	Moxifloxacin	No data	Liver	No Corticosteroids, survival
Um et al. [26]	42/F	Tazobactum moxifloxacin	No data	No data	No data
Nam et al. [8]	No data	Moxifloxacin	No data	No data	No data
Nori et al. [27]	23/F	Moxifloxacin	Nausea, vomiting, abdominal pain, rash	Liver, kidneys	Immunoglobulin, trachea intubation, boost, blood filtration, liver transplantation, dead
Son et al. [21]	50/F	Moxifloxacin	Fever, rash	Liver, lungs	No Corticosteroids, survival
Müller et al. [28]	44/M	Moxifloxacin associated with vancomycin and meropenem	Fever, rash	Kidneys	Corticosteroids, survival
Gohy et al. [29]	20/M	Rifampicin and moxifloxacin	Fever, rash	Kidneys, liver, pericardium, pleural effusion	No corticosteroids, survival
Our case	47/F	Moxifloxacin	Fever, cough	Liver, lungs, pleural effusion	No corticosteroids, survival

Moxifloxacin is widely used in respiratory infections and has a broad spectrum of activity against many microorganisms as a new generation fluoroquinolone antibiotic [30]. Although moxifloxacin-associated side effects such as recurrent tendonitis [31], interstitial nephritis [32] and anaphylactoid reactions [33] have been reported, DRESS syndrome induced by moxifloxacin is very rare. We report a case of a 47-year-old woman who developed DRESS syndrome with rare pulmonary involvement after receiving moxifloxacin treatment.

## Case presentation

**Fig. 1:**
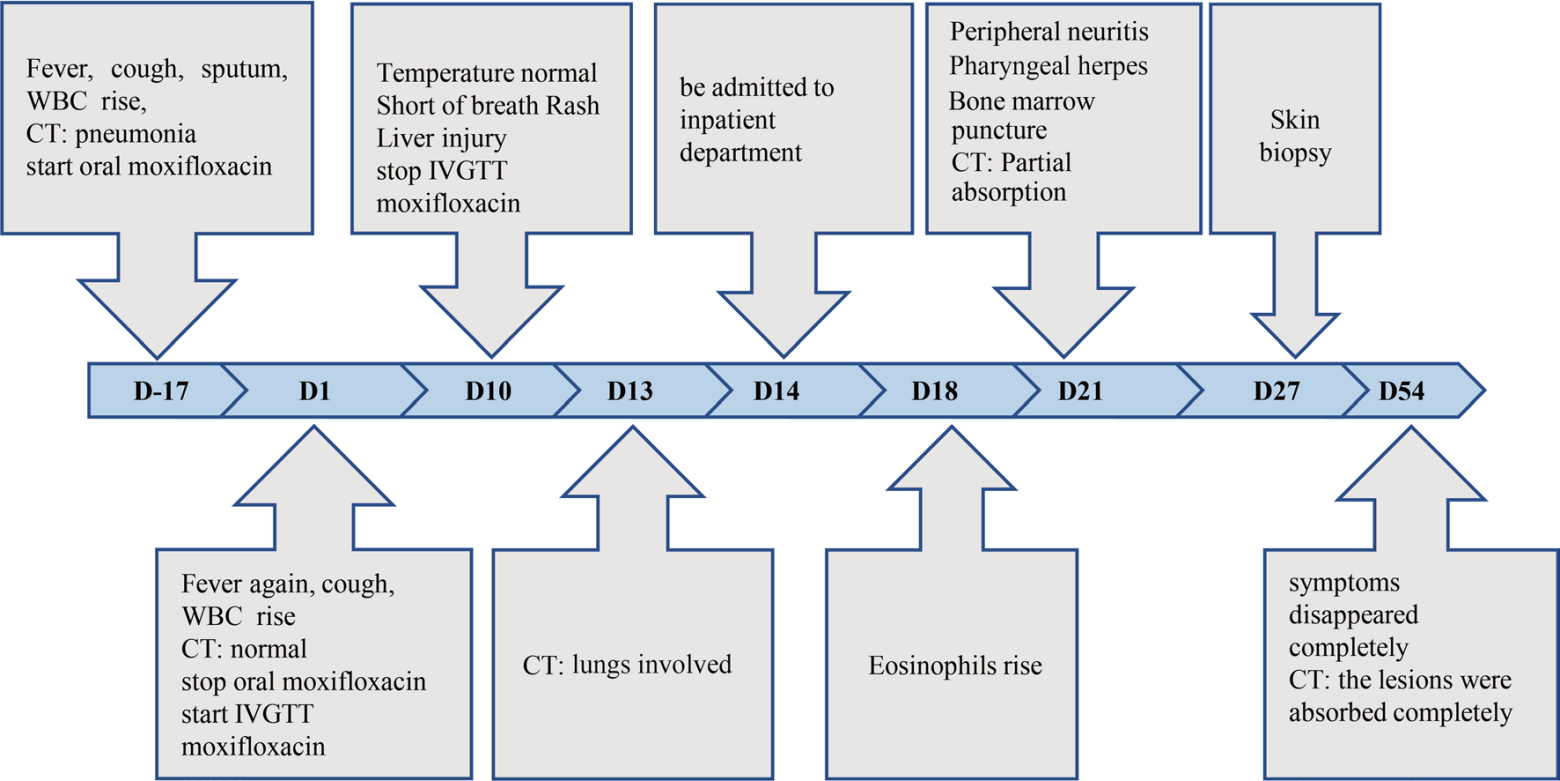
Timeline summarizing the symptoms and treatment of the patient

A 47-year-old woman presented to our department with cough and fever for 1 day. The diagnostic and treatment process of the entire course of the disease is shown in Fig. 1. She had a past medical history of hypertension, depression and type II diabetes. Her home medications included telmisartan, deanxit, and sertraline for 2 years and metformin for 1 year. She had allergies to cefotaxime-sulbactam, as the skin test was positive. She was recently diagnosed with community-acquired pneumonia. She was started on oral moxifloxacin 17 days prior to admission. On physical examination at admission, she was noted to be febrile (38.6 °C) and had a cough. There was no evidence of rashes, renal injury, lymphadenopathy, hepatosplenomegaly or pneumonia. The chest CT scan (D1) in the emergency department was normal and revealed that the previous inflammation in the left lower lobe (D-17) was resolved. Her laboratory tests were suggestive of infection, with a leukocyte count of 11,370/mm^3^ (neutrophils: 81.1%) and an absolute eosinophil count of 20/mm^3^ (within the normal range). She was admitted for possible bronchitis and placed on parenteral moxifloxacin. Six days after starting treatment with parenteral moxifloxacin, these laboratory tests were retested. However, her symptoms did not improve. Her temperature fluctuated between 37.2 and 37.9 °C during the treatment. Her cough and sputum production did not improve, and she then gradually developed shortness of breath.

**Fig. 2:**
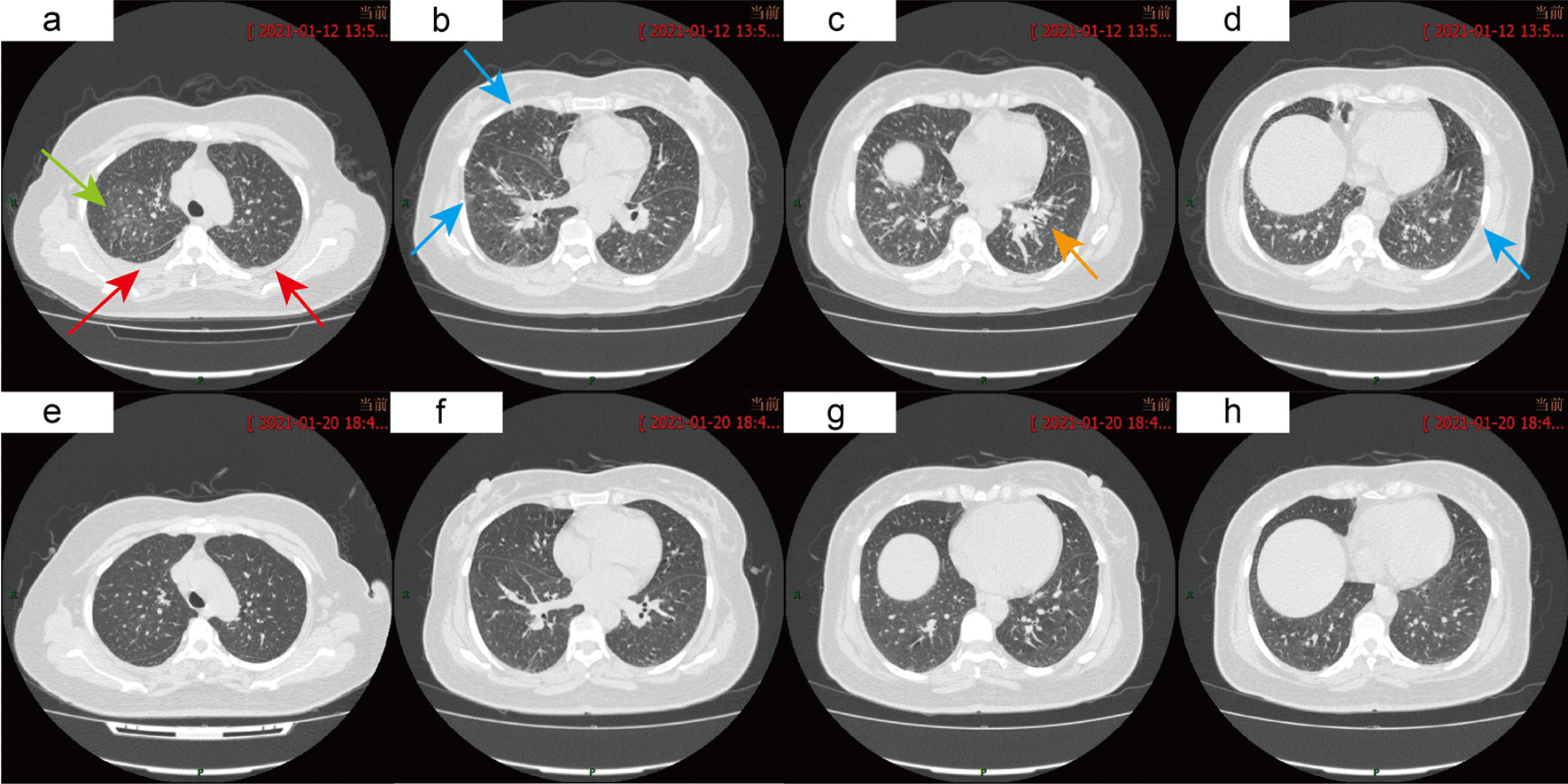
Changes in the chest CT (**a**–**d** are on D13: random pulmonary nodules (green arrow), a small amount of bilateral pleural effusion (red arrows), multiple areas of ground glass opacity (blue arrows), thickening of bronchovascular bundles (orange arrow); **e**–**h** are on D21: radiological improvement after withdrawal of the drug.)

**Fig. 3:**
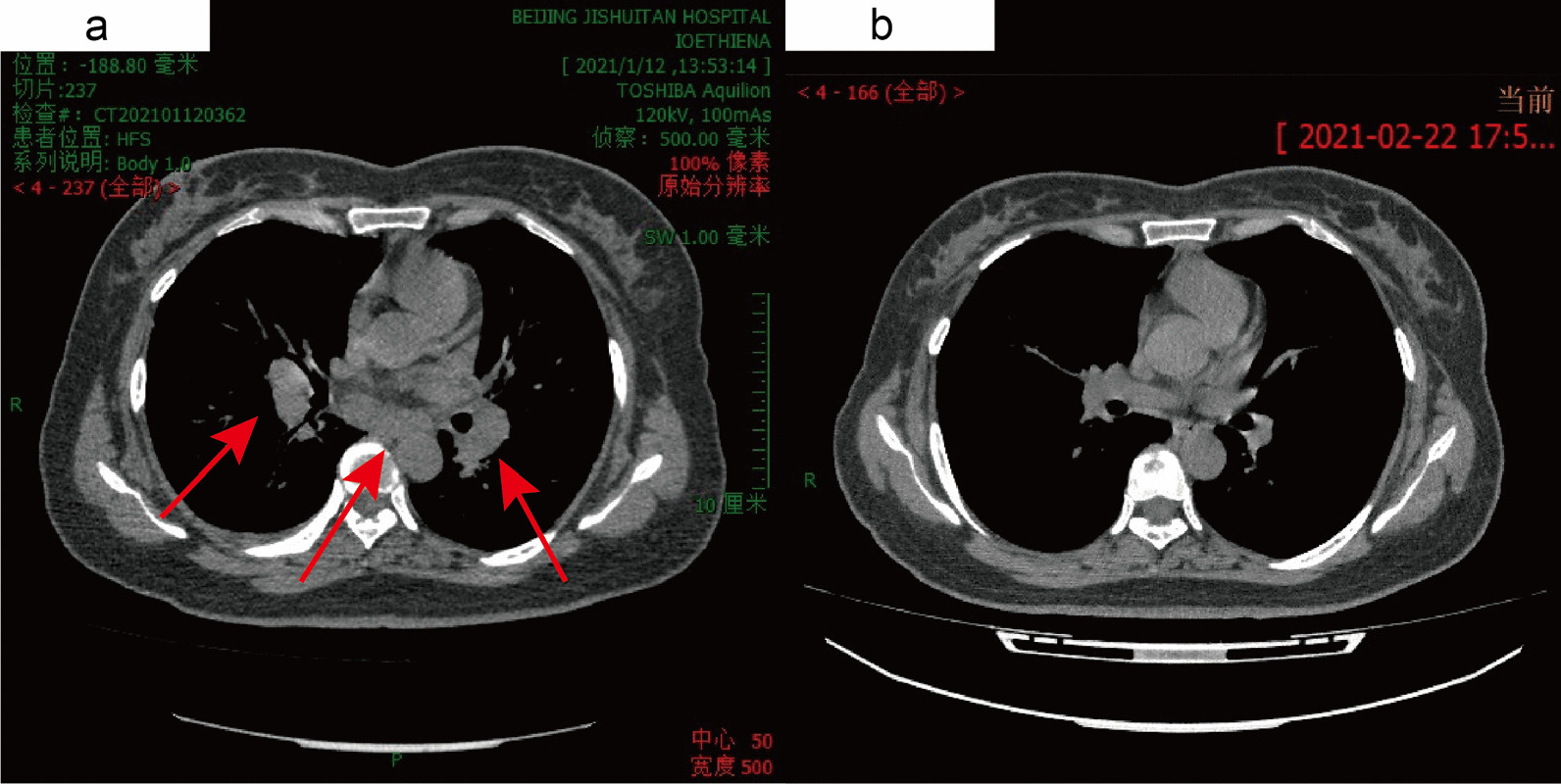
Changes in the lymph nodes at the mediastinum and hilar areas (**a** is on D13: mediastinal and hilar lymph nodes are larger than 1 cm; **b** is on D54: mediastinal and hilar lymph nodes returned to normal after withdrawal of the drug)

Nine days after treatment with parenteral moxifloxacin, she was referred to the Department of Rheumatology and Respiratory Medicine because of unexplained fever and shortness of breath. Chest CT (Figs. 2a–d, 3a, D13) showed random pulmonary nodules, a small amount of bilateral pleural effusion, multiple areas of ground glass opacity, thickening of bronchovesicular bundles, bilateral hilar and mediastinal lymphadenopathy. Laboratory tests showed possible liver disease with ALT (alanine amino transferase) at 304 IU/l (7–40), AST (aspartate amino transferase) at 82 IU/l (13–35), ALP (alkaline phosphatase) at 425 IU/l (35–100), TBIL (total bilirubin) at 42.3 µmol/l (5.1–19), DBIL (direct bilirubin) at 26.1 µmol/l (0–7), and γGT (γ-glutamyl transferase) at 981 IU/l (7–45). The workup for autoimmune diseases was negative and included myeloperoxidase antibody (< 20 IU/ml), proteinase 3 antibody (< 20 IU/ml), anti-CCP < 7 U/ml, HLA-B27 (-), ANA-IIF < 1:100, and ANA + ENA antibody spectrum (-).

**Fig. 4:**
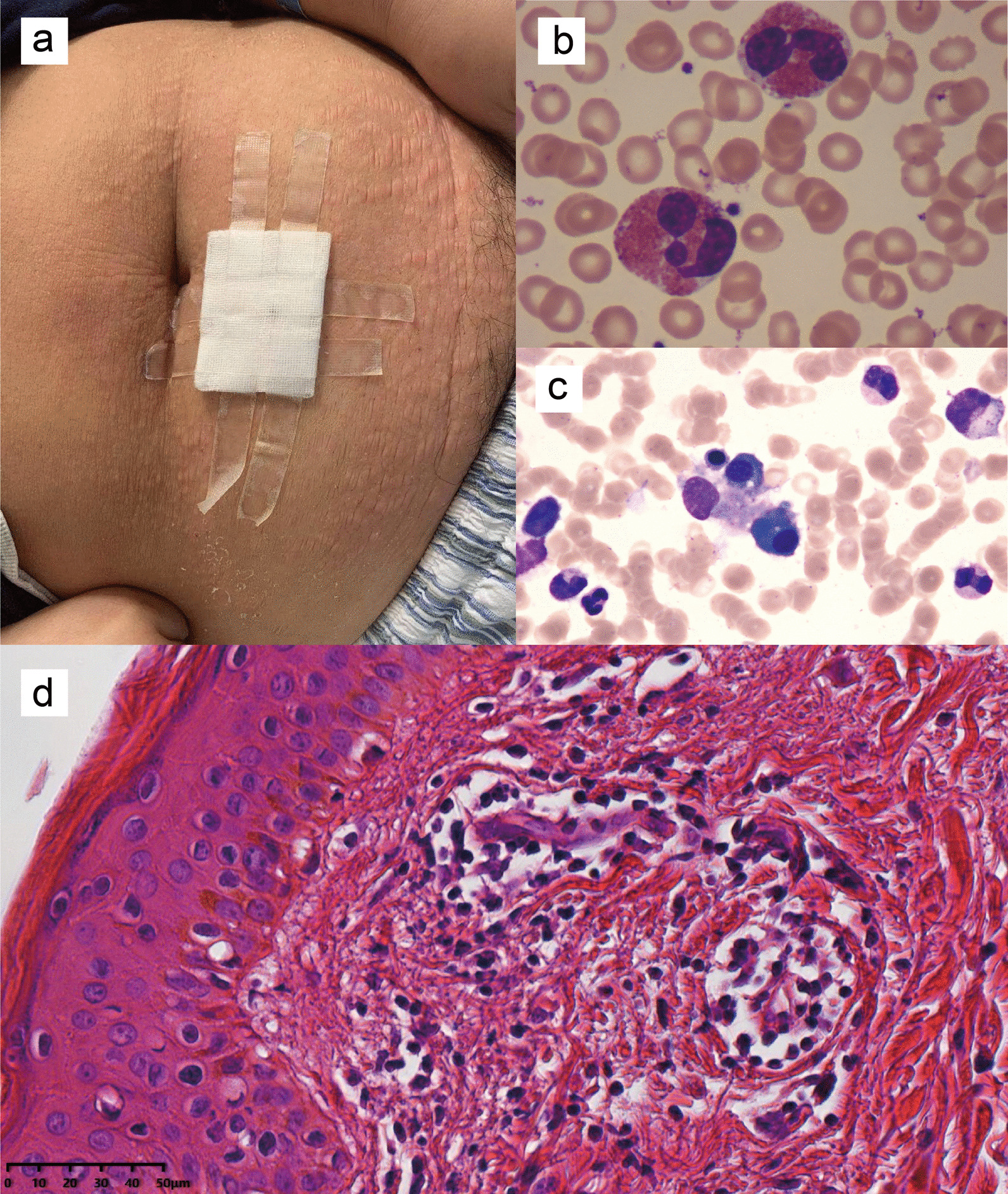
Characteristics of DRESS syndrome induced by moxifloxacin (a is rash on the abdomen; b is peripheral blood cell image analysis: Leukocytes showed reactive changes and the proportion of eosinophils was increased (36%); c is the bone marrow cell morphological analysis that showed 1% atypical lymphocytes; d is the skin biopsy from the abdominal area: the epidermis was mildly keratinized, lymphocytes were infiltrated around the small blood vessels in the superficial dermis, collagen fibers were proliferated)

Ten days after treatment with parenteral moxifloxacin, she developed a new morbilliform rash on her chest, abdomen, back, waist, buttocks and bilateral thighs. The rash faded under pressure, and the rash was accompanied by itching (Fig. 4a). Then, treatment with parenteral moxifloxacin was stopped.

Four days after parenteral moxifloxacin was stopped (D14), she was admitted to the inpatient department. After admission, routine laboratory examinations were completed. Blood gas analysis without oxygen inhalation showed hyperventilation and hypoxemia (pH 7.445, PCO_2_ 27.8 mmHg, PO_2_ 74.9 mmHg, HCO_3_ 19.3 mmol/l). Routine blood examinations showed that leukocytes were significantly increased (16,020/mm^3^). Peripheral blood cell image analysis showed that the leukocytes had reactive changes and that the proportion of eosinophils was increased (36%) (Fig. 4b). Approximately 2% of the lymphocytes were atypical. To rule out blood diseases, we performed bone marrow puncture, and the bone marrow cell morphological analysis showed that 1% atypical lymphocytes were present (Fig. 4c).

The patient had screening tests for infection: serology for hepatitis A, B, C, HIV and syphilis were negative. Sputum acid-fast staining and sputum culture were both negative. PCR testing of pathogens in the sputum was negative. Respiratory pathogen antibody testing was negative (including IgM of respiratory syncytial virus, adenovirus, influenza virus type A, influenza virus type B, parainfluenza virus, *Mycoplasma pneumoniae*, *Chlamydia pneumoniae*, *Pneumophila Legionella* type 1–7). Cytomegalovirus IgM antibody was negative, cytomegalovirus IgG antibody was 257.7 IU/ml (< 0.5). Antibodies and PCR for Epstein–Barr virus were negative.

The supplemental autoimmune antibody screening was negative, including mitochondrial antibody subtypes (AMA-M2, AMA-M4, AMA-M9) and anti-liver and kidney microsomal antibodies. The total IgE level was normal (3.09 U/l).

Her electrocardiogram was normal, and her echocardiography was also normal, with an ejection fraction of 64%. Multiple oval lymph nodes could be seen on both sides of her groin on B-ultrasound. The largest one on the left was 2.0 cm × 0.9 cm, and the largest one on the right was 2.3 cm × 0.9 cm. Pulmonary function tests (D18) (Table 2) were characterized by bronchial asthma with a positive bronchial relaxation test. In addition, it revealed a pattern of diffusion abnormality with decreased values of DLCO. They improved significantly after stopping moxifloxacin.

**Table 2:** Changes in pulmonary function

	D18	D29	D54
FVC/predicted value (%)	119	120	137
FEV1/predicted value (%)	111	113	127
FEV1/FVC (%)	75	76	75
DLCO/predicted value (%)	54	69	90
FEV1 increased (ml)^a^	> 200	< 200	< 200
FEV1 increased (%)^a^	19	1	2

^a^The drug used in the bronchial relaxation test is salbutamol sulfate 300 ug

**Fig. 5:**
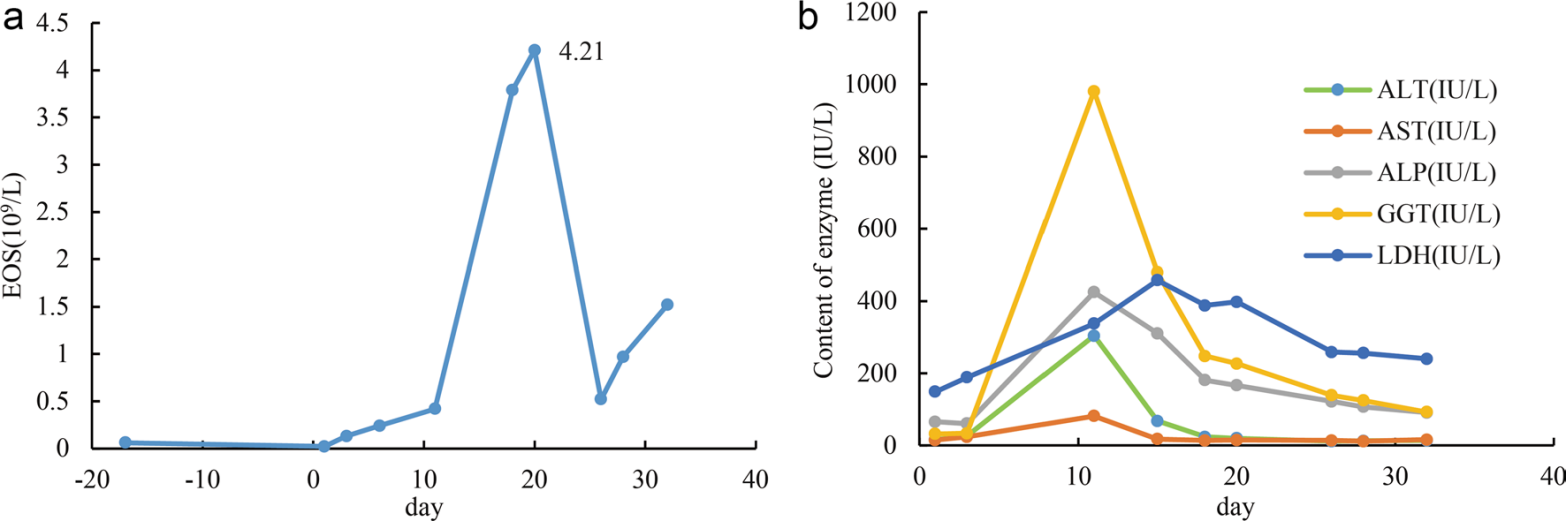
Changes in EOS and liver function (a shows changes in absolute eosinophils, and b shows changes in liver enzyme content)

After admission, the patient was given drug withdrawal and antiallergic and conventional liver-protecting treatment, which led to improvement. Therefore, systemic corticosteroid treatment was avoided in this patient. The changes in chest CT, routine blood tests, liver function, and pulmonary function tests were recorded (Figs. 2, 3, 4, 5, Table 2). On D21, CT showed that most of the lesions were absorbed (Fig. 2e–h). The patient developed pharyngeal herpes, which was clinically considered to be related to virus infection, and hyperesthesia in her distal extremities, which was clinically diagnosed as peripheral neuritis. However, testing for human herpesvirus has not been carried out since this institution has no detection capability. A skin biopsy was performed to exclude alternative diagnoses (Fig. 4d). On D54, the outpatient was rechecked, the symptoms were completely alleviated, and chest CT returned to normal.

## Discussion and conclusions

The patient in this case report did not take any new medications to cause the symptoms. There was no rash or fever during the patients’ long-term use of oral anti-hypotension drugs, anti-diabetic drugs and anti-anxiety drugs. Therefore, these drugs were less likely to be the etiology of DRESS in this patient. The offending drug that caused DRESS in this patient was likely used before the outbreak of the rash, which points to the only drug added—moxifloxacin. The rash appeared 27 days after starting moxifloxacin, with a latency consistent with that reported by Kardaun SH et al. [34]. The culprit drug in our case was identified by reviewing the medication history and clinical course (rash and systemic symptoms improved after stopping moxifloxacin). Peripheral blood counts, liver function tests, the determination of serum creatinine levels and urinalyses were performed to identify any internal organ abnormalities. The serological detection of autoantibodies and pathogenic detection were performed to exclude other autoimmune or infectious diseases. Other possible skin diseases were ruled out by consulting a dermatologist and obtaining a skin biopsy. The lymphocyte transformation test and patch testing were helpful in finding the culprit drug associated with the DRESS [35]. Unfortunately, these studies were not conducted in our case because the patient did not consent.

The exact mechanism of this syndrome is still unclear. It is believed that the drug-specific immune response induces virus reactivation and then activates cytotoxic CD8 + T lymphocytes, resulting in tissue damage [36]. Human herpesvirus (HHV), Epstein–Barr virus, and cytomegalovirus reactivation have been observed during the acute phase of DRESS syndrome, which has led to suggestions of a pathogenic connection [5]. PCR and serological antibodies against EBV were performed to exclude its activation. CMV-IgM antibody was negative, but CMV-IgG antibody was positive. The active state of CMV is unknown due to the lack of dynamic changes in CMV-IgG. Herpesvirus reactivation is also unknown limited by the detection ability of the institution.

Much work was done in this case to definitively diagnose DRESS syndrome in this case. Because of the different manifestations of DRESS syndrome and the involvement of different organs, a set of diagnostic criteria that can be easily applied in the clinic is needed. The RegiSCAR group has suggested a series of criteria to grade the possibility of DRESS syndrome. Our case is a definite case of DRESS syndrome according to the RegiSCAR group diagnosis scoring system because this case had a high score of 7 (Table 3).

**Table 3:** The RegiSCAR group diagnosis score for drug reaction with eosinophilia and systemic symptoms (DRESS)

	No	Yes	Unknown	Case
Fever (≥ 38.5 ℃)	− 1	0	− 1	0
Enlarged lymph nodes (≥ 2 sites, > 1 cm)	0	1	0	1^a^
Atypical lymphocytes	0	1	0	1^b^
Eosinophilia	0	1	0	
700–1499 × 10^6^/l or 10–19.9%		2		2^c^
≥ 1500 × 10^6^/l or ≥ 20%				
Skin rash	0		0	
Extent > 50%	0	1	0	1
At least two of: edema, infiltration, purpura, scaling	− 1	1	0	0
Biopsy suggesting DRESS	− 1	0	0	− 1^d^
Internal organ involvement	0		0	
One		1		
Two or more		2		2^e^
Resolution in > 15 days	− 1	0	− 1	0
At least three biological investigations done and Negative to exclude alternative diagnosis	0	1	0	1^f^

A schematic scoring system to grade the possibility of DRESS: < 2 no case; 2–3 possible case; 4–5 probable case; > 5 definite case [34, 37]. The last column represents the scoring for our patient, 7 points in total

^a^Inguinal lymph nodes on B-ultrasound and mediastinal/hilar lymph nodes on CT (Fig. 3a) > 1 cm

^b^Bone marrow cell morphological analysis showed atypical lymphocytes (Fig. 4c)

^c^ Absolute eosinophils were up to 4210 × 10^6^/l (Fig. 5)

^d^Biopsy of the skin was performed (Fig. 4d)

^e^Hepatic involvement: AST, total bilirubin, ALP all > 2UNL, once; pulmonary involvement: CT showed interstitial involvement (Fig. 2a–d) and abnormal blood gases

^f^Hepatitis A, B, C, mycoplasma/chlamydia, antinuclear antibody were negative

Lungs are rarely involved in DRESS syndrome. It typically starts with fever, and respiratory symptoms may precede the development of rashes. Pulmonary manifestations in DRESS syndrome are variable and nonspecific. The most common lung manifestations are interstitial pneumonia and pleural effusion. Lobar infiltration and pulmonary nodules have also been reported [9]. The differential diagnosis of DRESS syndrome with pulmonary involvement is complicated. A retrospective study reported that 45% of them were initially misdiagnosed as pneumonia and were empirically treated with antibiotics [9]. It may aggravate the condition, especially for those induced by antibiotics. Appropriate diagnosis and treatment require a shrewd physician who can recognize the symptoms of drug exposure and exclude other diagnoses.

The lung manifestations of our patient varied (Figs. 2 and 3). (1) Diffuse micronodules in both lungs, with random distribution overall: This is different from the centrilobular distribution in DRESS syndrome reported by Sawata et al. [38], which is a common pattern in hypersensitivity pneumonitis. The chest CT in our case showed that some nodules were in the pleural surface and interlobar fissure. It suggests the involvement of pulmonary interstitium. The typical random micronodules pattern signals an infectious or metastatic hematogenous distribution of disease [39]. However, the cause of the imaging pattern in our case may be complicated and remains to be elucidated. (2) There are multiple areas of ground-glass opacity under the pleura (Fig. 2b and d). This pattern indicates drug-induced eosinophilic pneumonia [40]. (3) The thickening of bronchovascular bundles (Fig. 2c) and bilateral pleural effusion (Fig. 2a): This is the imaging manifestation of pulmonary edema. Non-cardiogenic pulmonary edema is a common clinical manifestation of drug-induced pulmonary diseases. The pathogenetic mechanism of drug-induced NCPE (non -cardiogenic pulmonary edema) needs further studies. The pathogenetic mechanism of drug-induced non-ALI/ARDS NCPE involves allergic reactions which cause capillary leak syndrome (Clarkson syndrome), hypervolemia, and anaphylaxis [41]. The imaging patterns of drug-induced lung injuries (DLI) was classified as diffuse alveolar damage (DAD), chronic interstitial pneumonia (CIP), eosinophilic pneumonia (EP), organizing pneumonia (OP) and hypersensitivity pneumonia (HP), which can reflect the pathological pattern to some extent and help us evaluate prognosis [40]. Based on the chest CT, the disease type of our case should be a non-DAD pattern involving HP and EP, which indicates relatively favorable prognosis compared with DAD pattern.

Pulmonary function tests of DRESS syndrome with pulmonary involvement are rarely recorded in the literature. The tests revealed diffusion abnormalities, which may be caused by interstitial infiltrates. It is worth noting that it suggested bronchial smooth muscle spasm with positive bronchial relaxation tests and this performance disappeared after drug withdrawal. It is well known that the recruitment and activation of eosinophils are involved in the occurrence of bronchial asthma [42]. The results of this examination may suggest that eosinophils are involved in lung damage in DRESS syndrome. This has been preliminarily confirmed by histopathology. Hase I et al. found infiltration of eosinophils on histopathology of the transbronchial lung biopsy specimen besides CD8 + T lymphocytes in DRESS syndrome. [43].

DRESS is a rare syndrome. It is important to diagnose the disease as early as possible and provide timely treatment to improve the prognosis and reduce the mortality rate. The treatment measures include discontinuation of suspicious drugs and supportive care, such as the correction of electrolyte disturbances and nutritional support. For severe cases, systemic use of corticosteroids is a widely accepted treatment protocol [44].The DRESS syndrome mortality rate reported by a previous retrospective study was 5% to 10%, and most patients can fully recover from the disease. It has been reported that after the occurrence of DRESS syndrome, patients are more likely to develop autoimmune diseases such as systemic lupus erythematosus, autoimmune thyroiditis, rheumatoid arthritis and type 1 diabetes. Based on Taweesedt et al.’s suggestion [9], patients with internal organ involvement need to receive systemic corticosteroids. However, liver and pulmonary involvements of this case were mild. This case gradually recovered due to timely withdrawal of the culprit drug. The use of systemic corticosteroids was avoided. The occurrence of long-term complications needs further observation.

It is difficult to make a diagnosis in time because of the long latency, unpredictability, and complex and nonspecific manifestations. It is necessary to improve many tests to prevent other diseases. Information on DRESS syndrome involving the lungs caused by MX is scarce because few cases have been reported before. Moxifloxacin has been commonly used for respiratory infections. It is important to distinguish DRESS syndrome caused by drugs from aggravated infection so that proper treatment can be given to improve the prognosis of patients when the CT images and clinical symptoms worsen during drug use. This case is important for the differential diagnosis of DRESS syndrome and helps us to further understand the mechanism of DRESS syndrome.

## Data Availability

The datasets used and analyzed during the current study are available from the corresponding author on reasonable request.

## Abbreviations

DRESS: Drug reaction with eosinophilia and systemic symptoms
SOB: Shortness of breath
CT: Computed tomography
RegiSCAR: Registry of severe cutaneous adverse reaction
DIHS: Drug-induced hypersensitivity syndrome
HHV: Human herpesvirus
IgE: Immunoglobulin E
AGEP: Acute generalized exanthematic pustulosis
SJS: Stevens-Johnson syndrome
TEN: Toxic epidermal necrolysis
WBC: White blood cells
IVGTT: Intravenously guttae
ALT: Alanine amino transferase
AST: Aspartate amino transferase
ALP: Alkaline phosphatase
γGT: γ-Glutamyl transferase
TBIL: Total bilirubin
DBIL: Direct bilirubin
CCP: Cyclic citrullinated peptide
HLA-B27: Human leukocyte antigen B27
ANA-IIF: Antinuclear antibody by indirect immunofluorescence test
ANA: Antinuclear antibody
ENA: Extractable nuclear antigen
HIV: Human immunodeficiency virus
PCR: Polymerase chain reaction
IgM: Immunoglobulin M
IgG: Immunoglobulin G
EB: Epstein barr virus
AMA: Anti-mitochondrial
FVC: Forced vital capacity
FEV1: Forced expiratory volume in the first second
DLCO: Diffusing capacity for carbon monoxide
pH: Potential of hydrogen
PCO2: Partial pressure of carbon dioxide
PO2: Partial pressure of oxygen
HCO3-: Bicarbonate ion
CMV: Cytomegalovirus
EOS: Eosinophil
NCPE: Non-cardiogenic pulmonary edema
ALI: Acute Lung Injury
ARDS: Acute Respiratory Distress Syndrome
DLI: Drug-induced Lung Injuries
DAD: Diffuse Alveolar Damage
CIP: Chronic interstitial pneumonia
EP: Eosinophilic Pneumonia
OP: Organizing Pneumonia
HP: Hypersensitivity Pneumonia
MX: Moxifloxacin
